# Investigating a structured diagnostic approach for chronic breathlessness in primary care: a mixed-methods feasibility cluster randomised controlled trial

**DOI:** 10.1136/bmjresp-2024-002716

**Published:** 2025-02-13

**Authors:** Gillian Doe, Jill Clanchy, Simon Wathall, Shaun Barber, Sarah A Edwards, Helen Evans, Darren Jackson, Natalie Armstrong, Michael C Steiner, Rachael A Evans

**Affiliations:** 1 NIHR Respiratory BRC Leicester, Department of Respiratory Sciences, University of Leicester, Leicester, UK; 2 Clinical Trials Unit, University of Leicester, Leicester, UK; 3 Clinical Trials Unit, Keele University, Newcastle-under-Lyme, UK; 4 NIHR Respiratory BRC Leicester, University Hospitals of Leicester NHS Trust, Leicester, UK; 5 Chair Hinckley & Bosworth Medical Alliance, Leicester, UK; 6 Department of Population Health Sciences, University of Leicester, Leicester, Leicestershire, UK

**Keywords:** Not Applicable

## Abstract

**Background:**

There is a need to reduce delays to diagnosis for chronic breathlessness to improve patient outcomes.

**Objective:**

To conduct a mixed-methods feasibility study of a larger cluster randomised controlled trial (cRCT) investigating a structured symptom-based diagnostic approach versus usual care for chronic breathlessness in primary care.

**Methods:**

10 general practitioner practices were cluster randomised to a structured diagnostic approach for chronic breathlessness including early parallel investigations (intervention) or usual care. Adults over 40 years old at participating practices were eligible if presenting with chronic breathlessness without an existing explanatory diagnosis. The primary feasibility outcomes were participant recruitment and retention rate at 1 year. Secondary outcomes included number of investigations at 3 months, and investigations, diagnoses and patient-reported outcome measures (PROMs) at 1 year. Semistructured interviews were completed with patients and clinicians, and analysed using thematic analysis.

**Results:**

Recruitment rate was 32% (48/150): 65% female, mean (SD) age 66 (11) years, body mass index 31.2 kg/m^2^ (6.5), median (IQR) Medical Research Council dyspnoea 2 (2–3). Retention rate was 85% (41/48). At 3 months, the intervention group had a median (IQR) of 8 (7–9) investigations compared with 5 (3–6) investigations with usual care. 11/25 (44%) patients in the intervention group had coded diagnosis for breathlessness at 12 months compared with 6/23 (26%) with usual care. Potential improvements in symptom burden and quality of life were observed in the intervention group above usual care.

**Conclusions:**

A cRCT investigating a symptom-based diagnostic approach for chronic breathlessness is feasible in primary care showing potential for timely investigations and diagnoses, with PROMs potentially indicating patient-level benefit. A further refined fully powered cRCT with health economic analysis is needed.

**WHAT IS ALREADY KNOWN ON THIS TOPIC**

- There are delays to diagnosis lasting years for many patients with long-term conditions commonly presenting with breathlessness. A structured symptom-based diagnostic intervention for breathlessness with early parallel investigations may lead to earlier diagnosis and treatments to improve patient outcomes; however, the clinical- and cost-effectiveness of such an approach is unknown.

**WHAT THIS STUDY ADDS**

- We demonstrated that a future cluster randomised controlled trial investigating a symptom-based structured diagnostic intervention for breathlessness is feasible. Our results show a symptom-based approach for breathlessness in primary care has the potential to reduce time to diagnosis, improve outcomes for patients and appears acceptable to patients and clinicians.

**HOW THIS STUDY MIGHT AFFECT RESEARCH, PRACTICE OR POLICY**

- Our feasibility results overall support a fully powered multicentre randomised trial to formally assess clinical- and cost-effectiveness of a structured diagnostic breathlessness pathway. If successful, this approach can be implemented into clinical practice with policy recommendations.

## Introduction

 Breathlessness is a common and distressing symptom with an estimated prevalence of 9–11% in the general population,^1 2^ increasing with age to 25% of people over the age of 70 years old.^3^ High healthcare use is associated with breathlessness in both primary and secondary care,^4^,^6^ and functional impairment from breathlessness is associated with reduced survival.^7^

Over half of the cases of chronic breathlessness are caused by cardiorespiratory disease,^8 9^ with clinical data relating to patients over the age of 40 indicating the most common causes are chronic obstructive pulmonary disease (COPD), heart failure (HF), obesity, anaemia and anxiety.^9 10^ These conditions may be diagnosed or excluded with investigations frequently available in a primary care setting.

Previous epidemiological studies from primary care have highlighted missed opportunities to diagnose conditions commonly presenting with breathlessness such as COPD and HF, with a large number of patients diagnosed in later stages of the disease or during hospitalisation.^11 12^ Evidence around misdiagnoses for COPD, asthma and interstitial lung disease (ILD)^13 14^ also indicates significant challenges in accurate and timely diagnosis for patients. Although there is a National Institute for Health and Care Excellence (NICE) clinical knowledge summary for investigating breathlessness and a National Health Service (NHS) England diagnostic pathway support tool for breathlessness advocating for a symptom-based approach (2023), neither specify whether to request in parallel or sequentially nor a short timeframe.^15 16^ Clinicians describe supporting an incremental approach to investigation aligning with disease-specific diagnostic guidelines.^17^ However, using a large UK primary care database (Clinical Practice Research Datalink (CPRD)), we have shown that 57% (57975/101369) of adults waited beyond 2 years for a diagnosis, but adults diagnosed within 6 months of presentation with breathlessness have a lower risk of hospital admissions and mortality compared with those waiting longer.^18^ Breathlessness has also been shown to cause a significant burden of ill health among individuals without a confirmed diagnosis.^19^

Our overarching hypothesis is that a symptom-based approach for diagnosis in primary care for chronic breathlessness, including a holistic suite of diagnostic investigations at the point of presentation, will lead to earlier diagnosis, earlier treatment and improved outcomes for patients. This approach may also reduce future healthcare contacts and hospitalisations. However, there is clinical equipoise with concerns about overinvestigation, overdiagnosis and potential increased associated costs.^20^

To investigate the clinical- and cost-effectiveness of a structured symptom-based diagnostic approach for chronic breathlessness, a large and potentially expensive multicentre cluster randomised controlled trial (cRCT) would be needed. We therefore conducted a feasibility study to inform design of a future trial. The main feasibility aims were:

1. To assess the feasibility of participant recruitment and retention rate to enable calculation of the number of general practitioner (GP) practices, cluster sizes and duration of the ultimate cRCT.
2. To better understand potential primary outcome measures for the future trial.
3. To understand any influence of the trial design on usual care.

## Methods

### Study design

We conducted a mixed-methods feasibility study of a multicentre cRCT to investigate a structured diagnostic approach versus usual care for chronic breathlessness in primary care (REC reference: 19/EM/0201). The protocol has been published^21^ and registered as a clinical trial (ISRCTN: 14483247). We report the study in accordance with Consolidated Standards of Reporting Trials (CONSORT) reporting guidelines (CONSORT 2010 statement: extension to cluster randomised trials).

The mixed-methods design uses a convergent parallel approach where the quantitative and qualitative data are collected simultaneously, analysed separately and then brought together to enhance interpretation of the results.^22^

### Participants

10 GP practices in Leicestershire, UK, were cluster randomised to a structured diagnostic approach (intervention) including early investigations or usual care. Participants were opportunistically recruited from primary care when they presented to their GP with breathlessness. Participant eligibility criteria were adults over 40 years old, breathless for at least 2 months and within the first two presentations for breathlessness with a healthcare professional. Participants were excluded if they had an existing diagnosis for breathlessness, were acutely unwell requiring hospitalisation or had an estimated prognosis of less than 1 year. An electronic template, triggered at the point of consultation by breathlessness Read codes or free text, was used to aid opportunistic recruitment.^23^ Participants were blinded to their study arm and provided with the following information: ‘*Your GP surgery has been put into a group at random to use certain guidance to help find the cause of your breathlessness*.’

### Intervention

The structured diagnostic approach included history and clinical examination alongside early investigations to be performed within one month in parallel (online supplemental figure 1): body mass index (BMI), spirometry, electrocardiogram (ECG), chest X-ray, full blood count, N-terminal-pro Brain Natriuretic Peptide (NT-proBNP) profile, anxiety and depression screening using the Patient Health Questionnaire—4 item (PHQ-4)^24^ and the General Practice Physical Activity Questionnaire.^25^ By excluding common causes quickly, this approach aims to facilitate clinicians to request the next step of investigations quickly, that is, full pulmonary function tests and high-resolution (HR) CT thorax where suspected ILD, or echocardiogram for HF after a raised NT-proBNP. The structured pathway used in the study was the output from stakeholder engagement including people with lived experience of breathlessness, the NICE breathlessness clinical knowledge summary guidance and the Breathlessness IMPRESS Tips for Clinicians guidance,^10 15^ described in more detail in the protocol paper.^21^

A diagnostic pathway document was provided for GPs to support a structured history and examination, and prompt the investigations.^21^ In order to ensure participants in the intervention group had all investigations, if they were not performed in primary care, they were completed by the research team.

### Control

The usual care group were asked to proceed with investigating the patient and their symptoms as per usual practice and were directed to the NICE clinical knowledge summary for breathlessness^15^ to standardise care.

### Outcome measures

The primary outcome was recruitment and retention rate in order to subsequently calculate recruitment rate per practice to provide an estimation of cluster number and sizes required for future trial design. Ten GP sites were considered an adequate number of clusters. All feasibility outcomes are described in table 1. Secondary outcome measures included number of investigations and diagnoses at 3 and 12 months, and time to diagnosis. Patient-reported outcome measures (PROMs) were collected at baseline, 6 and 12 months.

**Table 1:** All feasibility outcomes

Feasibility measures	Outcomes
Number of patients recruited per GP practice population size	Patient recruitment ranged from 1 to 11 patients recruited per GP practice.
Retention rate	Retention rate was 85% (41/48).
Number of participating GP practices vs the number approached	10 out of the 15 GP practices that were contacted agreed to participate in this study.
Time for GPs to screen for eligibility	It is anticipated that the amount of time taken for clinicians to read through the electronic template with patients would vary between clinicians and patients. The electronic template was designed with Patient and Public Involvement and a patient representative devised the wording to maximise clarity for patients. Qualitative data from clinicians and practice staff indicate that the time taken within the patient consultation is acceptable (table 5).
Number of eligible patients who agree to be approached by the research team vs total number of eligible patients	The proportion of eligible patients who agreed to be approached vs the total number eligible ranged from 12% (11/89) to 57% (13/23) across the GP practices.
Number and timing of investigations in the diagnostic pathway completed	The Intervention group had a median (IQR) of 8 (7–9) tests compared with 5 (3–6) tests in usual care within 3 months (figure 2A).
Acceptability of the research visit to the participants	Participants described finding the walk test (Incremental Shuttle Walk Test) harder than expected and that there were a lot of questionnaires to complete, some of which were difficult to understand. Patient quotes describing the acceptability of research visits are presented in table 5.
Data collected from Interviews regarding participant experience of the trial	All participants interviewed expressed taking part in the study as a positive experience. Patient quotes describing participants’ experiences of the research visit are presented in table 5.

GPgeneral practitioner

Physical outcome measures were also collected at baseline; collection methods are described in detail in online supplemental material and protocol paper.^21^ PROMs included health-related quality of life: the Chronic Heart Questionnaire (CHQ) self-report and EuroQol 5 Dimension 5-Level (EQ5D-5L); breathlessness: Dypsnoea-12, Multidimensional Dyspnoea Profile (MDP), Baseline Dyspnoea Index, Transition Dyspnoea Index (TDI) and the Medical Research Council (MRC) Dyspnoea scale; anxiety and depression using the Hospital Anxiety and Depression scores; patient knowledge and skills to manage their own health using the Patient Activation Measure. Participants were contacted up to three times for completion and return of their PROM questionnaires. Ease of use of PROM questionnaires was assessed by missing data and support required from the research team with follow-up questionnaires.

All outcome measures collected were part of the research visit. None of the results of these outcome measures such as the PROMs and physical outcome measures were available to the GP practice and therefore did not influence diagnosis.

### Semistructured interviews

Semistructured interviews were conducted with patients, clinicians and GP practice staff to understand their experiences of the diagnostic process for breathlessness and taking part in the study. The qualitative methods are described in detail elsewhere.^17^ In brief, interviews were conducted by one of two researchers trained in qualitative methods, recorded and transcribed verbatim. Qualitative data were analysed using thematic analysis.^26^ The qualitative data addressing the feasibility aim to better understand ‘usual care’ have been reported elsewhere.^17^

### Statistical analysis

Normality of the data was assessed using the Shapiro-Wilk test. Data are presented as mean (SD) or median (IQR). Exploratory data analysis was completed for the secondary outcome measures. The primary feasibility outcome measure was recruitment and retention rates. Recruitment rate was recorded as the proportion of participants who consented compared with the number of participants identified as eligible for the study. SPSS V.26 was used for statistical analysis. GraphPad Prism 9 was used for all figures presented.

Exploratory analysis was performed on time to diagnosis using survival analyses based on Cox proportional hazards modelling of time to diagnosis.

### Patient and public involvement

The concept for this study was developed from discussions between patients and clinicians about how to improve local pathways for breathlessness as part of a National Health Service (NHS) Improving Quality project with the aim to streamline and coordinate care to achieve earlier diagnosis for patients with chronic breathlessness. Patients highlighted the delays to diagnosis they had experienced and the delays to treatments such as medication and exercise rehabilitation. The basis for the structured diagnostic approach used in this feasibility study was the output from stakeholder engagement using Listening into Action methods.^27^ The process included GPs, community and hospital clinicians with cardiorespiratory background, managers, commissioners (payers) and patients with lived experience of chronic breathlessness. Members from the relevant NIHR Biomedical Research Centre Patient and Public Involvement (PPI) groups were embedded throughout the study design and conduct, providing specific advice including the duration of the research visits, patient-facing information, choice of outcome measures and balancing the time and burden of completing questionnaires. The wording for the electronic template to aid recruitment was developed by members of the PPI group.^21 23^ To demonstrate the value of fully embedding PPI throughout the study process, we codeveloped a short animation for dissemination. The animation was narrated by a member of the PPI team (online supplemental figure 2).

## Results

The results are presented here aligning with the three feasibility aims.

### Recruitment and retention

10 out of the 15 GP practices approached agreed to participate in the study. All practices approached had a patient population of 10 000 or greater, with an Indices of Multiple Deprivation quintile range of 1–5.

**Figure 1:**
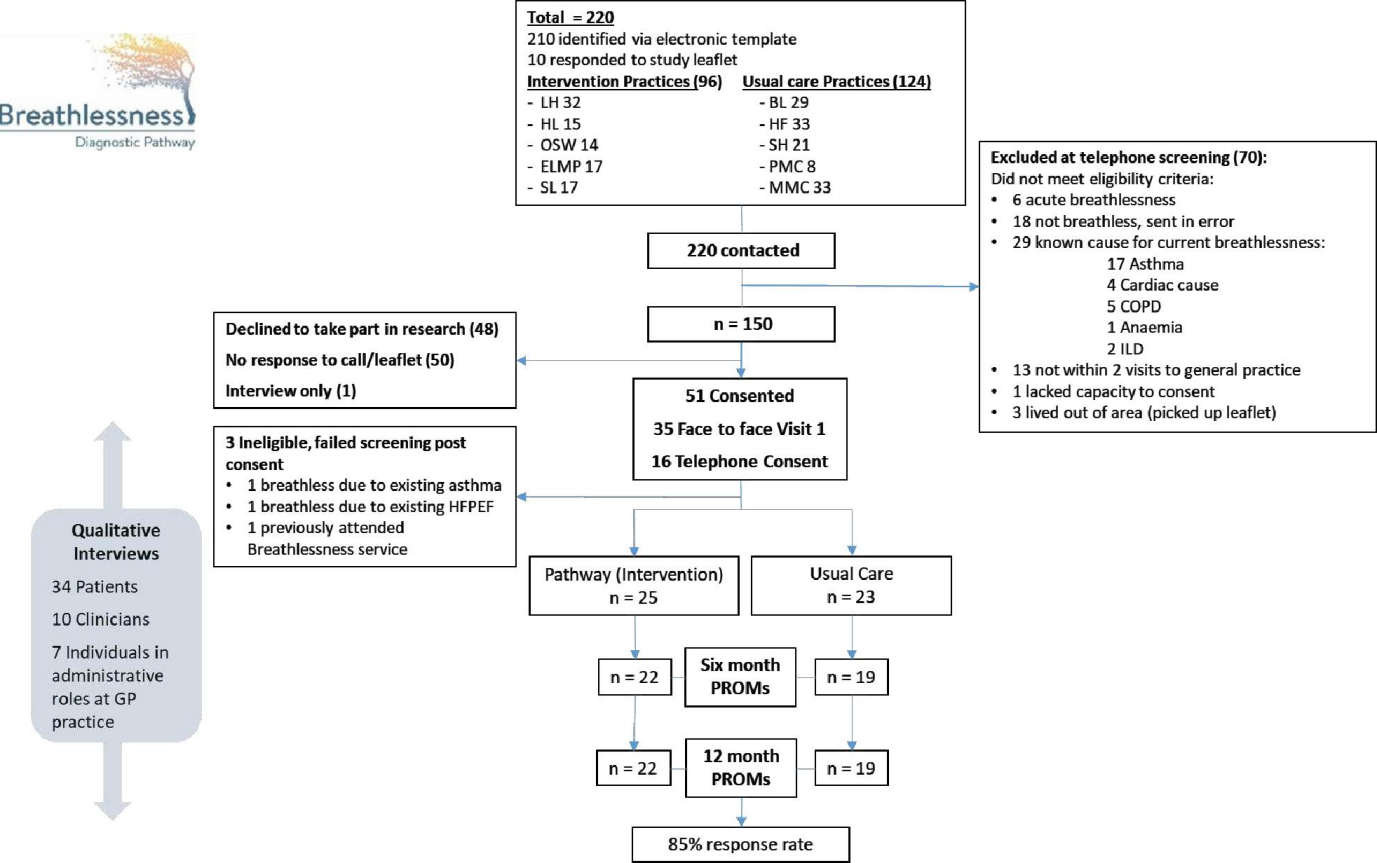
Consolidated Standards of Reporting Trials diagram. COPD, chronic obstructive pulmonary disease; GP, general practitioner; HFPEF, heart failure with preserved ejection fraction; ILD, interstitial lung disease; PROMs, patient reported outcome measures. *Individual Practices initialled rather than named.

Recruitment rate was 32%, with 48/150 participants recruited between November 2019 and February 2021 (figure 1): 65% female, mean (SD) age 66 (11) years, BMI 31.2 (6.5), median (IQR) MRC dyspnoea scale grade 2 (2–3) (table 2). The baseline characteristics between the intervention and usual care groups were similar (tables2  3 and online supplemental table 1). The intervention group had a slightly higher symptom burden in the baseline breathlessness PROMs compared with the usual care group. The recruitment rate ranged from 1 patient to 11 patients per GP practice population. The UK COVID pandemic started in March 2020. Recruitment rate pre COVID pandemic (study period November 2019 to March 2020) was 42%, with 36/86 participants recruited in this timeframe.

**Table 2:** Baseline characteristics

	All participants	Usual care	Intervention
	(n=48)	(n=23)	(n=25)
Age (years)	65.8 (11.3)	64.9 (11.6)	64.5 (11.3)
Gender, n (% female)	31 (65)	16 (70)	15 (60)
Ethnicity			
White	44 (92)	21 (92)	23 (92)
Asian/Asian British	2 (4)	1 (4)	1 (4)
Black/African/Caribbean/Black British	2 (4)	1 (4)	1 (4)
Body mass index (kg/m^2^)	31.2 (6.5)	30.8 (6.6)	31.7 (6.5)
Indices of Multiple Deprivation (quintile)	3 (2–4)	3 (2–5)	4 (3–4)
Medical Research Council dyspnoea score	2 (2–3)	2 (2–3)	3 (2–3)
Smoking status (%)			
Current	5 (10)	2 (9)	3 (12)
Former	21 (44)	11 (48)	10 (40)
Never	22 (46)	10 (43)	12 (48)
Pack years	16.0 (5.9–39.2)	16.0 (6.3–37.5)	16.0 (5.1–42.0)
-Range	0.2–120.0	0.2–47.0	0.25–120.0
Asbestos exposure—self-report (%)	9 (19)	7 (30)	2 (8)
Occupational dust exposure—self-report (%)	20 (42)	10 (44)	10 (40)
Living alone (%)	14 (29)	8 (35)	6 (24)
Retired (%)	28 (58)	14 (61)	14 (56)
Number of comorbidities	3 (2–6)	3 (1–5)	4 (2–7)
Number of medications	4 (0–5)	3 (2–6)	3 (1–6)
Rockwood frailty score	4 (3–4)	4 (3–4)	4 (3–4)

Data are presented as mean (SD), frequency (n, %) or median (IQR).

Dypsnoea scale. Rockwood frailty score: 1 = very fit, 2 = well no active disease, 3 = well with treated comorbid disease, 4 = apparently vulnerable, 5 = mildly frail, 6 = moderately frail, 7 = severely frail.

**Table 3:** Patient-reported outcome measures and physical outcome measures at baseline

	All participants	Usual care	Intervention
Patient-reported outcome measures	n=47	n=22	n=25
Dyspnoea −12	9.0 (3.0–17.0)	8.9 (2.0–13.5)	11.9 (4.3–17.5)
*MDP*			
A1	4.3 (2.4)	3.5 (2.3)	5.0 (2.4)
Immediate perception	19.0 (13.2)	14.9 (12.6)	22.5 (13.0)
Emotional response	15.2 (12.4)	11.7 (12.3)	18.3 (11.7)
*CHQ*			
Dyspnoea	3.2 (1.2)	3.2 (1.1)	3.1 (1.3)
Fatigue	3.7 (1.4)	3.7 (1.4)	3.8 (1.4)
Emotional	4.6 (1.3)	4.6 (1.4)	4.6 (1.3)
Function mastery	4.8 (1.4)	4.6 (1.3)	4.9 (1.6)
*HADS*			
Anxiety	7.2 (4.9)	6.6 (4.9)	7.7 (4.9)
Depression	5.6 (3.8)	6.1 (4.3)	5.3 (3.3)
EQ5D-5L VAS	70.1 (15.8)	66.8 (14.6)	74.6 (16.2)
EQ5D-5L Index Value	0.77 (0.64–0.85)	0.77 (0.67–0.85)	0.74 (0.43–0.84)
BDI focal score	6.4 (2.1)	6.2 (2.3)	6.7 (2.0)

Physical outcomes	n	% missing		n	% missing		n	% missing	
ISWT (m)	23	52	348 (196)	9	61	426 (217)	14	44	299 (170)
SpO2 post-ISWT (%)			92 (4)			93 (4)			92 (4)
Peak HR (bpm)			92 (18)			103 (17)			85 (15)
Peak BORG			3.0 (3.0–4.0)			3.5 (2.5–4.0)			3.5 (3.0–4.0)
SPPB (score)	31	35	9.0 (7.0–11.0)	11	52	8.0 (7.0–11.0)	20	20	9.0 (7.0–10.8)
4MGS (s)	31	35	4.0 (3.5–5.2)	11	52	3.9 (3.6–5.6)	19	24	4.1 (3.4–5.1)
Body fat (kg)	32	33	39.4 (9.0)	12	48	31.5 (10.0)	20	20	35.6 (13.0)
Body fat (%)			34.1 (12.0)			38.6 (9.9)			39.9 (8.7)
*Physical activity:									
Step count	32	33	5011 (2560)	11	52	5041 (3090)	21	16	4996 (2320)
Sedentary time (min)	29	40	660 (113)	12	48	649 (93)	17	32	668 (128)

47/48 participants completed the baseline PROMs. Physical outcome measures were collected where possible at face-to-face visits; some participants completed research visits by phone due to the pandemic. The number collected and % missing is presented.

Data are presented as mean (SD), frequency (%) or median (IQR).

* Physical activity: step count measured via Actigraph waist-worn device, Sedentary time measured via GENEActiv device.

A1affective domain 1 (relating to breathing discomfort)A2affection domain 2 (relating to emotional responses)BDIBaseline Dyspnoea Index (score from 0 to 12, with a lower score showing worse impairment)CHQChronic Heart Questionnaire (self-report)EQ5D-5LEuroQol- 5 Dimension 5 level questionnaireHADSHospital Anxiety and Depression ScoreHRheart rateISWTIncremental Shuttle Walk TestMDPMultidimensional Dyspnoea Index4MGS4 metre gait speedPAMPatient Activation MeasureSPPBshort performance physical batteryVASvisual analogue scale

Missing data are summarised in online supplemental table 2. All feasibility outcomes are described in table 1.

No serious adverse events were recorded for this study, and there were no safety concerns raised by participating patients or GP practices.

### Structured diagnostic approach versus usual care

**Figure 2:**
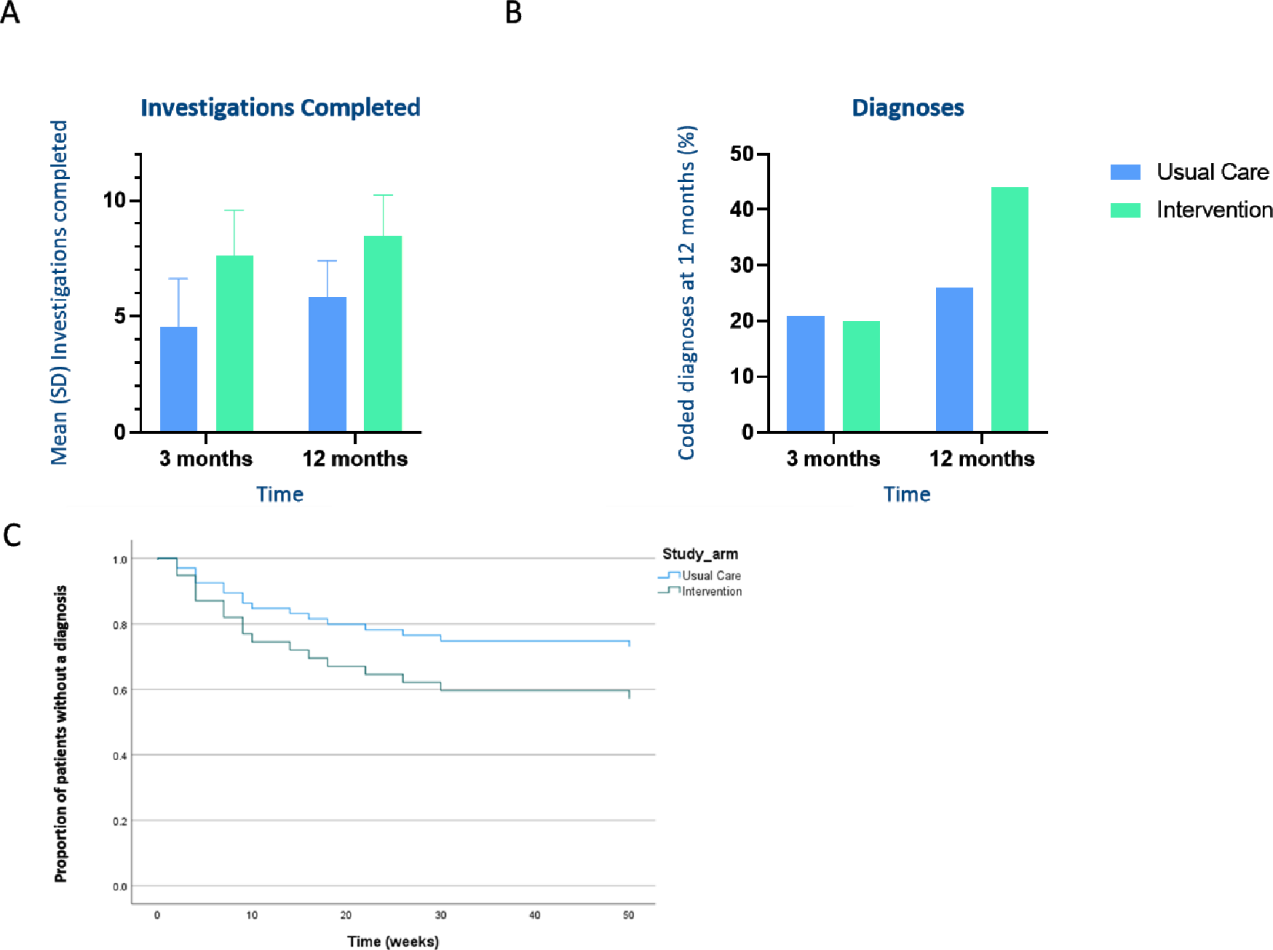
Investigations completed per patient and recorded diagnoses for intervention group versus usual care. (A) Mean (SD) number of investigations completed per patient at 3 and 12 months. (B) Proportion of coded diagnoses for breathlessness at 3 and 12 months. (C) Cox proportional hazards modelling of time to diagnosis over 1 year.

The intervention group had a median (IQR) of 8 (7–9) tests compared with 5 (3–6) tests in UC within 3 months (figure 2A). CXR, blood tests and BMI were the most frequently completed investigations in both intervention and usual care groups (online supplemental table 3). Spirometry was unable to be performed for periods of the study due to the COVID pandemic and the reason for non-completion was also recorded (online supplemental material).

At 12 months, 11 (44%) patients in the intervention group had a coded diagnoses for their breathlessness versus 6 (26%) patients in usual care (figure 2B). Coded diagnoses are summarised in online supplemental figure 3. Exploratory Cox proportional hazards modelling of time to diagnosis (figure 2C) derived a non-significant HR of 1.78 (95% CI 0.66 to 4.82, p=0.26), indicating the intervention group had 78% (95% CI −34% to 382%) greater chance of diagnosis relative to the usual care group.

Healthcare utilisation for both groups is summarised in online supplemental table 4.

For the PROMs (exploratory outcomes), the mean difference between the intervention and usual care groups from baseline to 12 months was greater than the minimal clinically important difference (MCID) for symptom burden and quality of life, the MDP immediate perception and emotional response domains, the Dyspnoea 12, the Dyspnoea domain of the CHQ and the Utility Index for the EQ5D-5L (table 4 and online supplemental figure 4). No difference between groups was seen in the Hospital Anxiety and Depression score. The TDI indicated an improvement in the intervention group and deterioration in the control group (online supplemental table 5).

**Table 4:** Comparison of patient-reported outcomes measures for 6- and 12-month responders

	Usual care (n=19)			Intervention (n=22)				
								Mean group difference
	Baseline	6 months	12 Months	Baseline	6 months	12 Months	MCID	(IG-UC) change from baseline at 12 months
MDP*								
Immediate perception	14.0 (12.9)	13.7 (14.0)	16.6 (15.2)	24.8 (12.1)	13.6 (10.6)	12.0 (10.1)	−4.6	−15.4 (3.5)†
Emotional response	9.8 (12.2)	8.4 (12.0)	10.9 (12.5)	18.6 (12.0)	11.9 (11.9)	11.7 (13.1)	−2.4	−8.5 (3.8)†
Dyspnoea-12*	7.4 (5.8)	9.4 (7.9)	9.1 (9.0)	12.7 (8.2)	8.7 (8.4)	8.1 (7.0)	−2.8	−6.3 (2.6)†
Nijmegen	16.3 (8.4)	17.8 (10.8)	15.6 (9.4)	22.2 (10.1)	19.2 (10.3)	17.5 (11.1)	N/A	−5.1 (2.6)
% with score indicating HVS	26	26	26	41	27	30		
CHQ								
Dyspnoea	3.3 (1.2)	3.6 (1.4)	3.7 (1.4)	3.0 (1.3)	4.5 (1.4)	4.3 (1.7)	0.5	1.0 (0.5)†
Fatigue	4.0 (1.3)	3.8 (1.4)	4.0 (1.3)	3.7 (1.5)	4.1 (1.4)	4.0 (1.4)	0.5	0.3 (0.3)
Emotional function	4.9 (1.4)	5.0 (1.4)	5.0 (1.4)	4.6 (1.2)	5.0 (1.3)	4.7 (1.5)	0.5	0.1 (0.3)
Mastery	4.9 (1.1)	5.0 (1.5)	5.1 (1.5)	4.8 (1.6)	5.1 (1.3)	5.2 (1.5)	0.5	0.2 (0.5)
HADS								
Anxiety	5.7 (4.1)	5.6 (4.6)	5.3 (4.3)	7.5 (4.5)	5.7 (3.1)	7.3 (4.5)	−1.7	0.3 (1.0)
Depression	5.6 (3.7)	5.3 (4.0)	6.1 (4.2)	5.3 (3.2)	4.7 (4.4)	6.0 (4.9)	−1.7	0.3 (1.0)
EQ5D-5L								
Index score	0.76 (0.16)	0.70 (0.33)	0.72 (0.25)	0.63 (0.31)	0.76 (0.20)	0.71 (0.26)	0.051	0.12 (0.07)†
VAS	68 (15)	66.3 (18.2)	67 (20)	74 (17)	74.30 (14.8)	67 (19)	6.9	−6 (5)
Patient Activation Measure	55.5 (10.9)	56.5 (12.9)	55.2 (9.3)	59.2 (14.7)	61.2 (15.6)	56.9 (15.5)	4	−1.2 (2.9)

Data are presented as mean (SD), frequency (%) or median (IQR). Shaded column is to highlight the minimally clinically important difference threshold for each of the outcome measures.

* MDP and Dypspnoea-12: reduction in score = improvement.

† Mean group difference (IG-UC) from baseline to 12 months greater than MCID.

CHQChronic Heart QuestionnaireEQ5D-5LEuroQol 5 Dimension 5 Level questionnaireHADSHospital Anxiety and Depression ScoreHVShyperventilation syndromeMCIDminimal clinically important differenceMDPMultidimensional Dyspnoea ProfileVASvisual analogue scale

From 82 questionnaire follow-ups (41 at 6 and 12 months), participants required help to complete with a range of 3–10 instances.

### Understanding the influence of the trial design

34 patient participants, 10 clinician participants and 7 GP practice staff completed semistructured interviews. Patients: 20 (59%) were female, mean (SD, range) age 68 (10.8, 48–89) years, 32 (94%) white British, 1 black African and 1 Asian British, median (IQR) indices of multiple deprivation quintile 3 (2–5). The clinicians had a mean (SD, range) of 17 (6.3, 6–30) years’ experience, 5 (50%) were female, 3 were Asian/Asian British and 7 were white British, 9 were GPs and 1 respiratory nurse. Six (86%) of the GP practice staff were female and all were white British. The qualitative data and themes addressing the feasibility aim to better understand ‘usual care’ have been reported elsewhere.^17^ The qualitative data related to study experience and influence of the study are presented in table 5.

**Table 5:** Feasibility measures collected from interviews with patients and clinicians

Time for GPs to screen for eligibility using the electronic template pop up (clinician and GP practice admin staff quotes)	“*Well, I have to say it’s been very unobtrusive hasn’t it. Because all that you’ve been asking us to do is ask the patient.”* (Clinician) “*I think on SystmOne as soon as you type breathlessness all of the information comes up which is really great. I think it prompts people to think about the study and to think about, is this patient possibly suitable?”* (Clinician) “*There’s a few GPs that get irritated by too many pop-ups, so I’ve had the odd comment about it. But I think that’s sometimes more a reflection of just the general stress and tiredness that everyone’s feeling at the moment more than anything.”* (Practice staff) “*But yeah when it popped up, not a problem … so it’s a fairly straightforward would you be interested or not?”* (Clinician) “… *since COVID, everybody’s breathless, so it popped up more times than it probably should have, because obviously more people are becoming more breathless with COVID and things like that. But before that I think it worked pretty well, because* *it’s just like a little reminder to the GPs to ask if they want to participate.”* (Practice staff)
Acceptability of the research visit to patients (patient quotes)	‘*I was very interested in it and I was very pleased to do the exercises and that to see, so that somebody else could see how good or bad I was, you know, with my breathing and that.’* (Patient)‘*It was all right once I was there and I did the tests, it was all right once I got back. It was a long day though’* (Patient)‘*Because you’ve taken time to explain things. Because there was a lot of good clear information sent out at the start.’* (Patient)‘*I had loads of forms, and I’m dyslexic, you helped me all through that though.’* (Patient)
Participant experience of the trial (Patient quotes)	“*I found it helpful, probably found it helpful just to talk as well, you know, to be able to talk to somebody about it [breathlessness]; instead of just, I suppose instead of just worrying about it, you know.”* (Patient)“*I think it’s been quite good. And I think it helps people to offload a bit as well, and think that somebody’s taking notice. I think that’s really important. To think that somebody’s actually going and researching and trying to make a difference, that’s important, especially if it’s like me when they think doctors aren’t listening, and thinking how important it is and how much it’s affecting people’s lives.”* (Patient)“*Well I think from my perspective lovely, because somebody’s interested in what I’m doing. But as far as the study goes, I’ve not found it intrusive or difficult … made to feel as though they’re valued and important. So yeah I think it’s a good thing. I’m interested in what you’re doing.’* (Patient)“… *you can talk about things that you wouldn’t normally talk about, to be fair, I mean, I wouldn’t say what I’ve just said to you to anybody else, to anybody, because the doctors don’t want to know that, understandable but no.”* (Patient)
GP experience of participating in the trial and influence on their practice (clinician quotes)	“… *the thought crossed my mind as to whether or not if I would do things differently. But no I don’t think it has because I think I would still do what I think is right for the patient … I was fairly confident that how we manage things in the practice I think we practice a good level of medicine, so I think I don’t mind the fact that we weren’t put into the intervention trial. So, I think that didn’t bother me really.”* (Clinician)*“From my point of view, I would possibly say no [to influence on practice], but that’s just myself, because I don’t think there’s anything really that I wouldn’t have already done in terms of investigation, how I’ve managed these patients. I suppose the only, thinking about it is I know in your study you’ve got the questionnaires haven’t you, the more mental health side questionnaires. I suppose whether that side of it, I’m more, I guess maybe more aware of that being a potential source of patient symptoms, the anxiety side of things.”* (Clinician)*“I think that does make you think about what you’re doing more. I mean from a, you know, you try not to change what you’re doing but I suppose you’re a little bit more cautious…you probably make a little bit more of an effort to write things more clearly and be a bit more thorough.”* (Clinician)*“It’s perhaps just thinking about how we teach GPs to approach it generally and about how we code breathlessness and what approach we take.”* (Clinician)

GPgeneral practitioner

All participants interviewed reported that taking part in the study was a positive experience. Participants commented there were a lot of questionnaires to complete, some of which were difficult to understand. One participant had additional needs to complete the questionnaires and the researcher supported them to ensure the questionnaires remained answered.

Clinicians and practice staff were mostly satisfied with the experience of being in the study. Views about using the electronic template for opportunistic participant recruitment were largely positive, in particular the low burden on time in a consultation, and are described in more detail elsewhere.^23^ The role of the GP practice to recruit patients appeared to be acceptable and interviewees expressed that although overall they did not feel being in the study influenced their practice in usual care, it made them more aware of the contributing factors to breathlessness (such as anxiety) and the need to be clear in their documentation.

### Future sample size

Using an SD of 1.61 from the CHQ Dyspnoea domain in this study, the MCID for the CHQ of 0.5,^28^ and with loss to follow-up of 15%, the sample required for 80% power at the 5% significance level before inflating for clustering is 386 participants (193 per arm); 328 overall before accounting for loss to follow-up. With an estimate of the intracluster correlation coefficient (ICC) of 0.1, derived from the participant data in this study from the CHQ Dyspnoea domain, the inflation factor is around 1.69 (based on mean cluster size of 7, minimum 2, maximum 12 after loss to follow-up; ICC=0.1), so the total sample required would be 658 randomised (329 per arm from 47 clusters); 556 overall before accounting for loss to follow-up.

## Discussion

Our overarching aim is to improve the symptoms, quality of life and survival for adults living with chronic breathlessness through earlier diagnosis using an affordable approach for the healthcare system. We report the first intervention study aiming to improve the time to diagnosis for patients living with breathlessness. Through a feasibility study, we show that a future cRCT investigating a structured diagnostic approach for breathlessness is feasible in primary care, demonstrated by a 32% recruitment and 85% retention rate. Our results indicate that the proposed symptom-based investigative approach, with parallel completion of early investigations rather than the usual incremental approach,^16 17^ supports the potential to reduce time to investigations and diagnoses for patients. The patient-reported outcomes indicated potential patient-level benefit with this approach, including symptoms and quality-adjusted life-years (EQ5D-5L at 1 year).

A primary outcome measure for a future trial could be a measure of health-related quality of life favoured by our patient and public involvement group. The CHQ was designed as a disease-specific health-related quality-of-life questionnaire and has been used in clinical trials for breathless populations.^29^ Using data from our trial, we have shown a future sample size calculation would include a manageable study size across 40 general practices (cluster unit). The EQ5D-5L utility index transformed into quality-adjusted life-years can be used for cost-effectiveness analysis,^30^ and our data would indicate potential responsiveness to the intervention.

Delays to diagnosis for people presenting with breathlessness are well documented, and we have recently shown in a cohort study that 33% of 101 369 patients did not receive any diagnosis within 2 years of presentation with breathlessness.^18^ We also reported that delays to diagnosis were associated with a higher risk of hospital admissions and mortality in the subsequent two years.^18^ Our symptom-based approach used in the current study contrasts with current disease-specific clinical algorithms for assessment and diagnosis described in a review^31^ whereby a stepwise approach is used for investigation and in a ‘disease silo’ from other potential contributing conditions. Many of the studies identified used patient history, physical examination, FBC, CXR and ECG as the first stage in their diagnostic algorithm but without a specified timeframe. Importantly only one study in the review was undertaken in primary care, highlighting the lack of evidence despite primary care being the most likely place of first presentation with breathlessness with cross-sectional data suggesting breathlessness accounts for 4% of consultations in primary care.^5^

NHS England have developed and recently published a diagnostic tool for breathlessness^16^ which closely aligns with the diagnostic investigations used in the current study, but did not advocate performing an early panel of investigations, rather it provides flexibility to complete initial investigations according to clinical judgement. However, our data from CPRD highlights the current delays to diagnosis and associated worse outcomes from the latter approach. Our qualitative research conducted as part of this mixed-methods study highlighted the possible reasons for delay to diagnosis included challenges with symptom recognition, timely investigations and confirming a positive diagnosis.^17^ An incremental approach to investigation to rule out individual diagnoses was described by clinicians, aligning with disease-specific guidelines which promote excluding a particular diagnosis, rather than a holistic approach to find all causes of a symptom. Following an incremental approach could be appropriate if timely investigations and multiple reviews were possible; however, this is commonly not achievable, would use more clinician time and has been further exacerbated by the COVID-19 pandemic causing delays in healthcare.^32^ A symptom-based approach also enables identification of multiple causes of breathlessness which is important and relevant as the prevalence of multiple long-term conditions rises and is a major problem for healthcare systems.^33^ There remains clinician equipoise between using an early parallel investigations approach versus sequential investigations, but our study supports the former and a larger trial is feasible.

Our research also raises the question of which other investigations could be included in a diagnostic pathway in primary care with a desirable criterion of being low cost, accessible, sensitive and specific. We found that a holistic approach to breathlessness was often absent and screening for anxiety and depression was not routinely recorded as part of usual care with only 8% assessed in the usual care group. Even in the intervention group, anxiety and depression screening was frequently picked up at the research visit having not been completed in primary care. A common screening tool is the four-item PHQ-4 screening tool, and this can be routinely embedded in electronic patient healthcare systems. Given the high prevalence of anxiety and depression associated with breathlessness,^9^ it is important to include screening as part of the diagnostic approach in the breathless patient population.^34^ We also need to consider the impact of breathing pattern disorder (BPD) as a cause for breathlessness and a future diagnostic approach may need to include assessment for BPD. Tools to assess BPD include the Breathing Pattern Assessment Tool and the Nijmegen questionnaire which can screen for hyperventilation, but neither are commonly used in primary care and BPD diagnosis often requires specific clinician expertise. BPD has become more commonly seen in primary care due to long COVID.^35^

Asthma was the most common diagnosis in both groups of the current feasibility study despite the population being over 40 years old. We purposefully chose not to include fractional exhaled nitrous oxide (FeNO) in our panel of investigations due to the diagnostic approach of having all investigations for all patients. FeNO is a relatively quick and easy investigation to complete in primary care, with NO as a biomarker of type II airway inflammation commonly seen in the diagnosis of asthma.^36^ It has been shown to have good specificity for diagnosing asthma, particularly in the presence of wheezing and rhinitis, but a lower sensitivity.^37^ We made an assumption that the population over 40 were at high risk of the common conditions our panel were able to either diagnose or exclude and tested whether doing all the investigations as a panel led to further diagnoses. An early panel of investigations will also capture multiple diagnoses for breathlessness facilitating a treatable traits personalised medicine approach to encompass mental health support, weight management and promotion of physical activity. Further refining the approach to add individual risk stratification for chronic cardiorespiratory disease might help reduce any unnecessary investigations. Similarly, increasing the complexity of the pathway to include assessment of the pre-test probability of asthma would help suggest how FeNO testing should be integrated. We only tested rather basic investigations in the current study, but still showed the potential for a positive signal for the majority of outcomes. Research is ongoing to understand the risk factors for breathlessness using machine learning that could also be added to a future algorithm.^38^

Advances are being made with other biomarkers other than blood tests; the role of exhaled breath volatile organic compounds in differentiating acute breathlessness has been explored as an option for non-invasive diagnostics in acute settings with cardiorespiratory patients.^39^ It is not currently known how this could translate into primary care but there is an urgent need for novel diagnostics, particularly for airways disease.

While we focused on performing simple, basic investigations which should be readily accessible in primary care, notwithstanding the challenges with spirometry,^40^ we acknowledge this is only the first step in the diagnostic process. However, even by influencing the investigations at this early step, 44% of patients in the intervention group of the cRCT had a coded diagnosis for their breathlessness at 12 months compared with 26% in usual care. These investigations (and the time saved by doing them early and in parallel) should help in selecting further investigations and/or specialist reviews. A further feature of our approach locally is a joint cardiorespiratory specialist clinic for unexplained breathlessness after the panel of investigations. Patients in both clusters could have been referred to this clinic by their GP and therefore may have reduced the comparable effect of the intervention. We only used ‘coded diagnoses’ to reflect the healthcare record used by clinicians.

We have previously reported from patient interviews that breathlessness management is an unmet need while awaiting a diagnosis and others report the wider patient unmet need in those with an established diagnosis.^17^ Although this was a feasibility study, symptom burden and quality-of-life outcomes in our study indicate possible patient benefit for those in the intervention group, but we acknowledge the importance and necessity of specific breathlessness self-management and therapies including exercise rehabilitation in addition to the diagnosis and disease-specific treatment.

More females than males were recruited to the study and reflects the baseline population; in a community population survey for breathlessness (1) there was a higher prevalence of breathlessness in women (11.3%) than men (6.3%; p<0.001, OR 1.9; 95% CI 1.5 to 2.4), and (2) data from a cross-sectional study showed 66% of a middle-aged population presenting with breathlessness were female (9).

### Strengths and limitations of the study

Due to the study recruitment period, it is anticipated that the pandemic and the subsequent impact on primary care processes may have reduced the number of patients presenting to their GP,^32 41^ willingness to participate in the study and availability of some of the diagnostic tests, particularly spirometry. Spirometry was halted entirely in primary care from March 2020 to the end of our study period.^42^

The intervention group appeared to have slightly better health reflected in the baseline measures compared with the usual care group. The recruitment rate of 32% (48/150) was considered acceptable based on previous studies 43,45 and was achieved despite the known challenges of recruiting in primary care and with the added restrictions resulting from the COVID-19 pandemic. The recruitment rate prior to the COVID pandemic of 42% is likely more relevant for future trial design, and the range in recruitment seen between practices indicates the need to account for this variation within the future trial sample size calculation. Our study employed a pragmatic approach with the intervention embedded in clinical care at the GP practice level, opportunistic recruitment at the point of patient presentation and adaptation to the design allowing continued recruitment through the COVID pandemic.

Opportunistic recruitment was a successful approach in this study design to identify a patient by a symptom at presentation in order to intervene in real time and has been shown to be of benefit in previous primary care studies.^46^ There are many identified barriers to recruiting to research in primary care, including insufficient funding, resource and research experience in GP settings,^44^ and recruitment rates typically vary in primary care research with other primary care trials reporting comparable rates of 25%^47^ and 42%.^46^ Our work has demonstrated that signposting patients about research at the point of presentation to healthcare, while reducing the burden on clinicians to discuss the research in detail, is a helpful approach.^23^

Cluster randomisation at the level of the GP practice was selected to reduce the risk of contamination in usual care, and this appeared successful. The proposed diagnostic tool and future trial design might require further refinement. Of note, most participants recruited were of white British ethnicity which is not representative of the diverse ethnic backgrounds of our local population. Further work is needed to ensure diverse patient and public engagement and representation is embedded in the future trial design.^48^

## Conclusion

Our results indicate that a cRCT investigating a symptom-based structured diagnostic approach for chronic breathlessness is feasible in primary care, with recruitment rates comparable with fully powered definitive trials in primary care. Improving patient care and experience for those living with breathlessness requires prompt and accurate diagnosis, allowing access to appropriate treatment and support. The structured diagnostic approach for chronic breathlessness used here appeared acceptable to patients and clinicians, with the potential to achieve more timely investigations and explanatory coded diagnoses, leading to potential patient-level benefit at 6 and 12 months. We report a positive indication that early parallel investigation as part of a structured diagnostic approach is of benefit but further refinement and a fully powered cRCT with health economic analysis would be needed to fully evaluate clinical and cost-effectiveness.

## supplementary material

10.1136/bmjresp-2024-002716 online supplemental file 1

## Data Availability

Data are available upon reasonable request.
