# Supplementary material for: Investigating a structured diagnostic approach for chronic breathlessness in primary care: a mixed-methods feasibility cluster randomised controlled trial
Source: BMJ Open Respir Res. 2025 Feb 13;12(1):e002716. doi: 10.1136/bmjresp-2024-002716 (PMC11831280; doi:10.1136/bmjresp-2024-002716)
Supplement: online supplemental file 1 [file bmjresp-12-1-s001.pdf]

.

Supplementary Material

Collection of Physical outcome measures

Physical outcome measures were collected for all participants attending an in-person research visit. Each participant's waist and hip circumference was measured and Body Mass Index (BMI, kg/m^2) was calculated by measuring the patient's height and weight. Body composition using bioelectrical impedance was measured to provide lean mass and body fat percentage. Exercise capacity was measured using the incremental shuttle walk test (ISWT) [1]. Frailty was measured using Fried's Frailty definition [2], the Rockwood Frailty scale [3], the Timed up and Go and the Short Physical Performance battery (SPPB) [4]. Physical Activity was assessed using the GT3x Actigraph device [5] and wrist worn GENEActiv device [6]. All outcome measures and data collection methods are described in detail in the protocol paper [7].

Spirometry

In the usual care group spirometry was not completed in 16 participants (13 not requested by clinician) In the intervention group spirometry was not completed in 11 participants, (5 not requested by clinicians). Some of this was due to the COVID pandemic.

Supplementary Table 1. All Physical Outcome measures collected at baseline and 12 months

	All Participants				Usual Care				Intervention			
	n	Baseline	n	12 months	n	Baseline	n	12 months	n	Baseline	n	12 months
ISWT (m)	23	348 (196)	14	451 (158)	9	426 (217)	6	522(161)	14	299 (170)	8	399 (143)
SpO2 post-ISWT (%)		92 (4)		93 (3)		93 (4)		95(2)		92 (4)		92 (4)
Peak HR (bpm)		92 (18)		102 (13)		103 (17)		112 (12)		85 (15)		94 (7)
Peak BORG		3.0 (3.0-4.0)		3.5 (2.7-4.8)		3.5 (2.5-4.0)		4.0 (3.5-4.3)		3.5 (3.0-4.0)		3.0 (2.3-3.8)
Reason for terminating test:												
Breathlessness				10 (71)				4 (67)				6 (75)
Pain				1 (7)				0				1 (12.5)
Breathless/Leg fatigue				2(15)				1 (16.5)				1 (12.5)
Other				1 (7)				1 (16.5)				0
QMVC (kg)	30	28.7 (15.1)	19	30.5 (14.6)	10	28.0 (14.8)	7	35.5 (16.4)	20	29.0 (15.6)	12	27.5 (13.2)
Handgrip (kg)	31	30.4 (11.8)	20	32.8 (13.5)	11	29.5 (4.3)	7	38.5 (15.6)	20	30.8 (2.4)	13	29.6 (11.8)
SPPB (score)	31	9.0 (7.0-11.0)	20	10.5 (9.0-12.0)	11	8.0 (7.0-11.0)	7	11 (10.0-12.0)	20	9.0 (7.0-10.8)	13	9.0 (8.0-11.5)
TUG (seconds)	30	8.9 (7.1-11.3)	20	8.2 (6.6-8.9	11	9.7 (6.4 -17.9)	7	6.9 (6.3-8.2)	19	8.9 (7.1-10.8)	13	8.4 (7.4-9.7)
4MGS (seconds)	31	4.0 (3.5-5.2)	20	4.0 (3.5-4.6)	11	3.9 (3.6-5.6)	7	3.6 (3.1-4.1)	20	4.1 (3.4-5.1)	13	4.3 (3.9-5.1)
Fried's Frailty:												
Robust	29	7 (24)	17	5 (29)	11	4 (36)	7	4 (57)	18	3 (17)	10	1 (10)
Pre Frail		20 (69)		12 (71)		5 (46)		3 (43)		15 (83)		9 (90)
Frail		2 (7)		0		2 (18)		0		0 (0)		0
Body fat (kg)	32	34.1 (12.0)	19	33.7 (13.3)	12	31.5 (10.0)	6	26.0 (6.7)	20	35.6 (13.0)	13	37.2 (14.3)
Body fat (%)	32	39.4 (9.0)	19	38.1 (8.5)	12	38.6 (9.9)	6	31.5 (7.3)	20	39.9 (8.7)	13	41.2 (7.4)
Physical Activity:												
Step count (Actigraph)	32	5011 (2560)	27	4753 (2853)	11	5041 (3090)	13	4772 (3034)	21	4996 (2320)	14	4735 (2790)
Overall Activity (mg)	29	21.8 (7.7)	26	20.2 (6.1)	12	21.0 (6.0)	13	18.7 (5.8)	17	19.5 (5.9)	13	21.7 (6.2)
**Time Inactive (mins)*	29	660 (113)	26	674.7 (63.5)	12	649 (93)	13	670.2 (65.8)	17	668 (128)	13	679.1 (63.5)

Data is presented as Mean (SD), frequency (%) or Median (IQR). *HR = heart rate, QMVC = quadriceps maximal voluntary contraction, SPPB = short performance physical battery, TUG = timed up and go, 4MGS = 4 metre gait speed*. *Time Inactive defined as time not moving including standing and therefore different to sedentary time (sitting, lying or reclining).

.

Feasibility of data collection

Thirty-two participants completed in person research visits at baseline as per the original study design, 16 completed their consent and research visit by phone. Of those who attended an in-person research visit, 23/32 (72%) completed the exercise capacity ISWT. Reasons for non-completion was elevated blood pressure beyond the acceptable limits for the walk test and mobility limited by pain. Forty-seven of the forty-eight participants recruited returned the PROM questionnaires at baseline.

A large proportion of the secondary outcome measures assessing physical function were missing from the 12 month follow up due to the change in study protocol which moved to remote data collection as a result of the pandemic (supplementary Table 2). Due to this incomplete data set of physical measures, only PROMs results are presented for comparison at six and twelve months to baseline in the manuscript. No serious adverse events (SAEs) were recorded for this study.

Ease of use of PROM questionnaires was assessed by missing data and support required from research team with follow up questionnaires. From 82 questionnaire follow ups (41 at six and twelve months), the CHQ required help from one participant compared with a range of 3 to 10 instances for other questionnaires.

Supplementary Table 2. Physical Outcome measures missing data at 12 months

Outcome measure	Not completed n	Usual care n (%)	Female n (%)
ISWT	30	15 (50)	21 (70)
QMVC	25	14 (56)	18 (72)
Handgrip	24	14 (58)	18 (72)
SPPB	24	14 (58)	18 (72)
TUG	23	14 (61)	18 (72)
4MGS	24	14 (58)	18 (72)
Bioelectrical impedance	25	15 (60)	18 (72)

44 Participants completed a follow up visit at 12 months; 20 in person and 24 by phone, 41 returned questionnaires.

.

Supplementary Table 3. Number and proportion of investigations completed

	3 months		12 months	
**Investigation**	Usual Care n = 23	Intervention n = 25	Usual Care n = 23	Intervention n = 25
Body Mass Index (BMI)	12 (52)	14 (56)	16 (70)	21 (84)
Chest X-ray	15 (65)	23 (92)	20 (87)	25 (100)
Electrocardiogram (ECG)	10 (44)	19 (76)	15 (65)	20 (80)
Spirometry	5 (22)	11 (44)	7 (30)	14 (56)
Full Blood Count (FBC)	18 (78)	21 (84)	20 (87)	23 (92)
NT-proBNP	13 (57)	20 (80)	16 (70)	21 (84)
Urea and Electrolytes	17 (74)	22 (88)	20 (87)	23 (92)
Thyroid Stimulating Hormone	13 (57)	19 (76)	17 (74)	20 (80)
PHQ-4	1 (4)	20 (80)	2 (8)	21 (84)
GPPAQ	1 (4)	18 (72)	1 (4)	20 (80)
All above	0	4 (16)	0	8 (32)
All above minus Spirometry	0	5 (20)	0	11 (44)

PHQ-4 = Patient health questionnaire 4 item, GPPAQ = GP Physical Activity Questionnaire.

Data presented as frequency (%)

.

Identify sources of data to plan the economic evaluation for a full trial

Primary care and hospital healthcare records were reviewed and data collected about number of GP consultations, referrals to secondary care, hospital outpatient appointments and hospital admissions (Table 4). An NHS Digital application to collect the healthcare utilisation data from Office for National Statistics (ONS) and Hospital Episodes Statistics (HES) was set up through the Data Access Request (DARS) portal; the appropriate data products to request were recorded and the information required has been mapped for a future, larger trial. We propose that this process would be required for a future trial and healthcare record review would not be feasible on a larger scale.

Supplementary Table 4. Healthcare Utilisation related to breathlessness

	**12 months**	
	Usual Care (n=23)	Intervention (n=25)
GP contacts	4 (3-5)	4 (2-4)
Referral:		
Respiratory	2 (9)	3 (12)
Cardiology	5 (22)	4 (16)
Breathlessness Service	1 (4.5)	3 (12)
Other	2 (9)	0
Coded Diagnosis	6 (26)	11 (44)
Outpatient appointments		
0	17 (73.1)	15 (60)
1	0	3 (12)
2	4 (17.2)	4 (16)
3	1 (4.3)	1 (4)
4	0	2 (8)
5	0	0
6	1 (4.3)	0
Hospital admissions	3 (12)	1 (14)

Data presented as frequency (%) or Median (IQR)

.

Supplementary Table 5. Baseline and Transition Dyspnoea Index

	All Participants		Usual Care		Intervention	
	n		n		n	
BDI focal score	48	6.4 (2.1)	23	6.2 (2.3)	25	6.7 (2.0)
TDI focal score 6 months	39	1.1 (3.7)	18	0.3 (3.2)	21	1.9 (3.9)
TDI focal score 12 months	44	0.6 (4.3)	21	-0.9 (3.7)	23	2.0 (4.5)

Data is presented as Mean (SD), *BDI = Baseline Dyspnoea Index (focal score ranges from 0-12 with 0 representing worse impairment), TDI = Transition Dyspnoea Index (focal score ranges from -9 to +9 with -scores being a deterioration and +scores being an improvement).*

Supplementary Figure 1. Investigations included in structured diagnostic pathway (Intervention)

Investigation	Implications for diagnosis
**Body Mass Index (BMI)**	To diagnose obesity if >30kg/m^2
**Electro cardiogram (ECG)**	To detect arrhythmias or diagnoses suggestive of ischaemic heart disease
**Chest X-ray**	To detect signs of heart failure or pulmonary pathology e.g. pleural effusion, interstitial changes
**Spirometry** (If FEV_1/FEV_6 <0.75 perform full spirometry)	Obstructive pattern (FEV_1/FVC < 0.70) may suggest airway disease such as COPD or asthma<br>Restrictive pattern (FEV_1/FVC > 0.70 but FEV_1 and FVC < 80% predicted) may suggest conditions such as obesity, interstitial lung disease, chest wall abnormality or neuromuscular disease
**Full Blood Count (FBC)**	Haemoglobin to exclude anaemia<br>Eosinophil count if >0.3 x10^9/L can support a diagnosis of asthma
**NT-pro BNP profile**	To exclude heart failure
**Urea and electrolytes/ TSH**	To exclude metabolic causes
**Activity Questionnaire GPPAQ**	To assess physical activity levels
**PHQ4**	Screening questionnaires for symptoms of anxiety and depression

FEV1; Forced Expiratory Volume in 1 second, FEV6; Forced Expiratory Volume in 6 seconds, NT-pro BNP; N-terminal brain natriuretic peptide, TSH; Thyroid stimulating hormone, GPPAQ; General Practice Physical Activity Questionnaire, PHQ4; Patient Health Questionnaire 4 item.

Supplementary Figure 2. QR code link to Breathe Deep patient and public engagement animation

(https://www.youtube.com/watch?v=SD9IsFhlJqA)

.

Supplementary Figure 3. Breakdown of coded diagnoses at 12 months

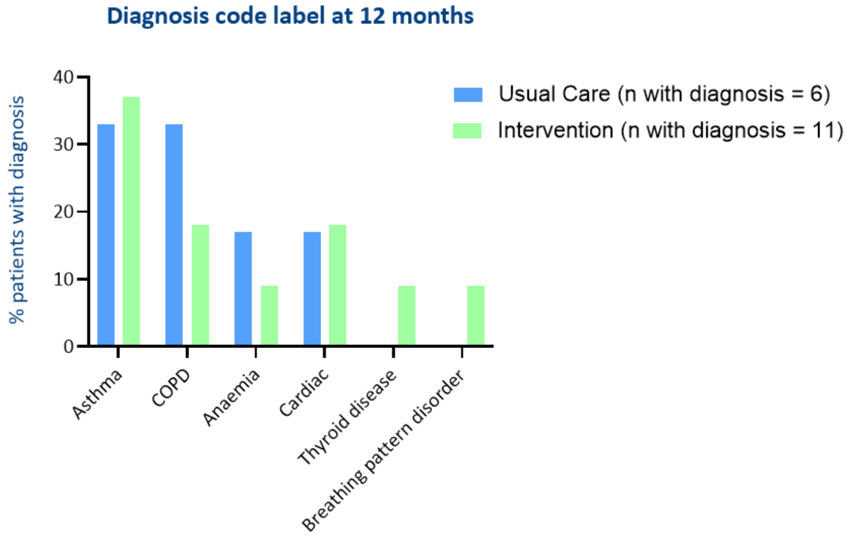

Figure 2 presents the breakdown of coded diagnoses for the participants who received a coded diagnosis at 12 months. In the Usual care group six patients had a coded diagnosis for their breathlessness; two were coded with COPD, two were coded with Asthma, one with Anaemia and one with Atrial Fibrillation (AF). In the Intervention group 11 patients had a coded diagnosis; four were coded with Asthma, two were coded with COPD, one with Cardiomyopathy, one with Anaemia, one with HF, one with an autoimmune Thyroid disorder and one with breathing pattern disorder.

Supplementary Figure 4. Patient reported outcome measures

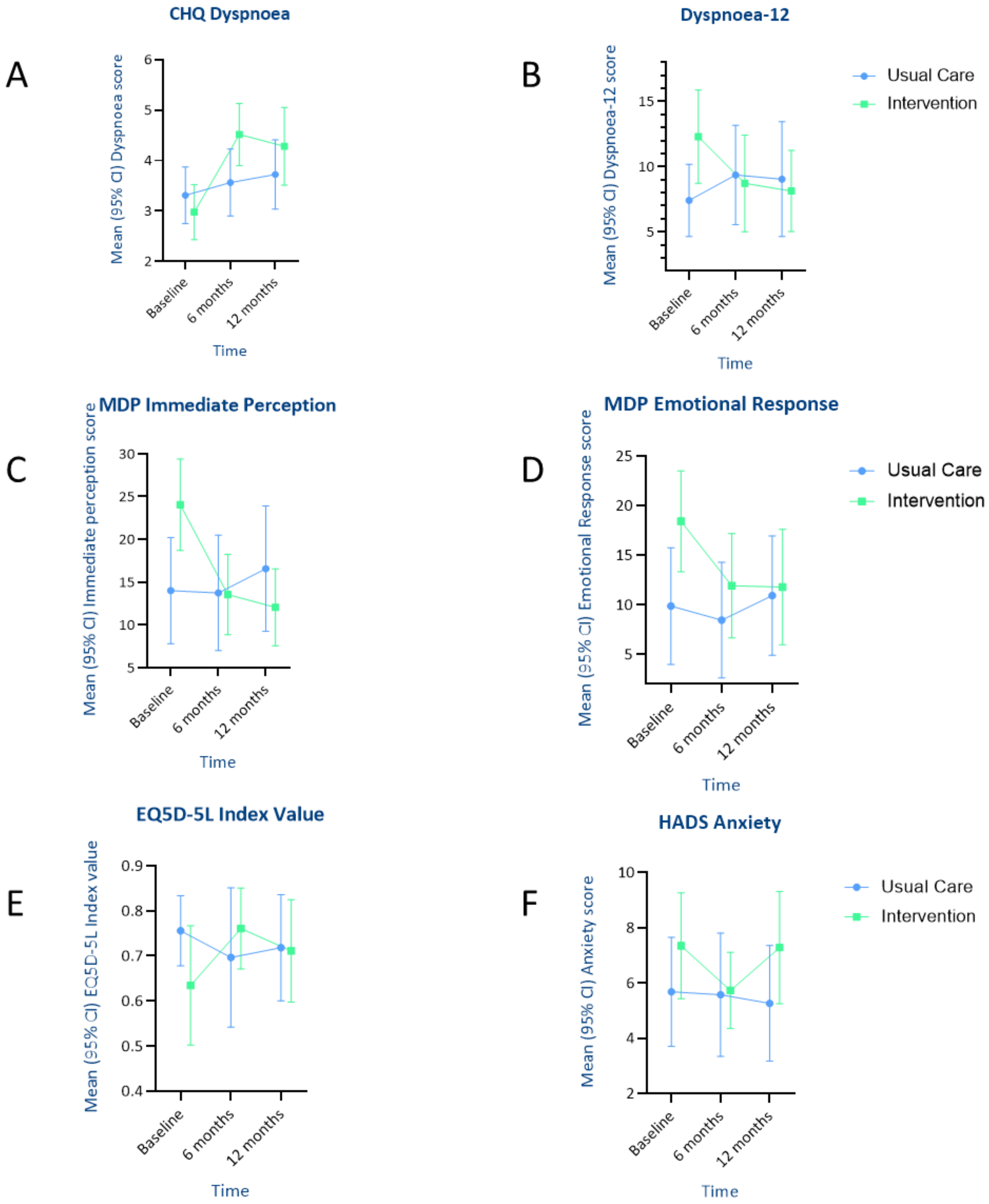

Mean score and 95% Confidence Interval (CI) for PROMs at baseline, 6 and 12 months. A. Chronic Heart Questionnaire (CHQ) Dyspnoea domain; B. Dyspnoea-12 questionnaire; C. Multidimensional Dyspnoea Profile (MDP) Immediate perception; D. Multidimensional Dyspnoea Profile (MDP) Emotional Response; E. EuroQoL 5 Dimension 5 level (EQ5D-5L) Index value score; F. Hospital Anxiety and Depression (HADs) questionnaire Anxiety score

.

References

1. Singh SJ, Morgan MD, Scott S, Walters D, Hardman AE. Development of a shuttle walking test of disability in patients with chronic airways obstruction. *Thorax* 1992: 47(12): 1019-1024.
2. Fried LP, Tangen CM, Walston J, Newman AB, Hirsch C, Gottdiener J, Seeman T, Tracy R, Kop WJ, Burke G, McBurnie MA, Cardiovascular Health Study Collaborative Research G. Frailty in older adults: evidence for a phenotype. *J Gerontol A Biol Sci Med Sci* 2001: 56(3): M146-156.
3. Rockwood K, Song X, MacKnight C, Bergman H, Hogan DB, McDowell I, Mitnitski A. A global clinical measure of fitness and frailty in elderly people. *CMAJ* 2005: 173(5): 489-495.
4. Guralnik JM, Simonsick EM, Ferrucci L, Glynn RJ, Berkman LF, Blazer DG, Scherr PA, Wallace RB. A short physical performance battery assessing lower extremity function: association with self-reported disability and prediction of mortality and nursing home admission. *J Gerontol* 1994: 49(2): M85-94.
5. Actigraph https://theactigraph.com/actigraph-wgt3x-bt
6. GeneActiv https://www.activinsights.com/actigraphy/geneactiv-original/
7. Doe G, Clanchy J, Wathall S, Chantrell S, Edwards S, Baxter N, Jackson D, Armstrong N, Steiner M, Evans RA. Feasibility study of a multicentre cluster randomised control trial to investigate the clinical and cost-effectiveness of a structured diagnostic pathway in primary care for chronic breathlessness: protocol paper. *BMJ Open* 2021: 11(11): e057362.
